# GSK-3β activation is required for ZIP-induced disruption of learned fear

**DOI:** 10.1038/s41598-020-75130-5

**Published:** 2020-10-26

**Authors:** Sukwoon Song, Jihye Kim, Kyungjoon Park, Junghwa Lee, Sewon Park, Sukwon Lee, Jeongyeon Kim, Ingie Hong, Beomjong Song, Sukwoo Choi

**Affiliations:** 1 School of Biological Sciences, College of Natural Sciences, Seoul National University, Seoul, Republic of Korea; 2 Department of Neural Development and Disease, Korea Brain Research Institute, Daegu, Republic of Korea; 3 Department of Psychiatry, McLean Hospital, Harvard Medical School, Belmont, MA 02478 USA; 4 The Solomon H. Snyder Department of Neuroscience, Johns Hopkins University School of Medicine, Baltimore, MD USA; 5 International Research Center for Neurointelligence (WPI-IRCN), The University of Tokyo Institutes for Advanced Study (UTIAS), The University of Tokyo, Tokyo, Japan

**Keywords:** Neuroscience, Physiology

## Abstract

The myristoylated zeta inhibitory peptide (ZIP), which was originally developed as a protein kinase C/Mζ (PKCζ/PKMζ) inhibitor, is known to produce the loss of different forms of memories. However, ZIP induces memory loss even in the absence of PKMζ, and its mechanism of action, therefore, remains elusive. Here, through a kinome-wide screen, we found that glycogen synthase kinase 3 beta (GSK-3β) was robustly activated by ZIP in vitro. ZIP induced depotentiation (a cellular substrate of memory erasure) of conditioning-induced potentiation at LA synapses, and the ZIP-induced depotentiation was prevented by a GSK-3β inhibitor, 6-bromoindirubin-3-acetoxime (BIO-acetoxime). Consistently, GSK-3β inhibition by BIO-acetoxime infusion or GSK-3β knockdown by GSK-3β shRNA in the LA attenuated ZIP-induced disruption of learned fear. Furthermore, conditioned fear was decreased by expression of a non-inhibitable form of GSK-3β in the LA. Our findings suggest that GSK-3β activation is a critical step for ZIP-induced disruption of memory.

## Introduction

Long-term potentiation (LTP) has long been postulated as a cellular mechanism of memory formation^1^, and most efforts to investigate this mechanism have been focused on elucidating the key molecules required for maintaining LTP. Several kinases have been implicated in LTP: Ca^2+^/calmodulin-dependent protein kinase II (CaMKII), mitogen-activated protein kinase (MAPK), protein kinase A (PKA), and protein kinase C (PKC)^2^. Each of these kinases is post-translationally activated upon the induction of LTP^3^, and inhibition of their activities impairs LTP induction^4–7^. However, it is unlikely that these kinases are critical in maintaining memory since their activities are of importance only within a limited period of time^4,6,8–14^.

On the other hand, Sacktor and his colleagues have suggested that protein kinase Mζ (PKMζ), a constitutively active PKC isoform, is required for maintaining L-LTP (late-phase LTP) and memory^15–17^. The amount of PKMζ persistently increases after LTP induction^18^, and blocking its activity using either chelerythrine or myristoylated zeta inhibitory peptide (ZIP), a specific peptide inhibitor of PKMζ, results in the reversal of L-LTP in hippocampal slices^15,18–22^. Consistently, it has been reported that PKMζ is required for maintaining many types of memory. Injection of ZIP or chelerythrine induces the impairments of spatial memory^16,21,23^, aversive taste-associated memory^24,25^, fear-conditioned memory^16,26^, instrumentally-conditioned memory^16^, sensorimotor memory^27^, object location memory^28^, and eyeblink-conditioned memory^22^.

Although several lines of evidence from both in vitro and in vivo studies support that PKMζ is required for maintaining LTP and memory, data from genetic approaches have challenged this hypothesis. In PKC/PKMζ-knockout mice, both L-LTP and long-term memory remain intact^29–31^. In addition, ZIP application in the knockout mice induces L-LTP reveral^29,31^ and memory disruption^29–31^ as it does in wild type mice. Intriguingly, in these studies, a scrambled version of ZIP (SCR-ZIP), which has been used frequently as a control drug^15,20,25,32^, also has effects on PKMζ activity, L-LTP and memory that are similar to those of ZIP, suggesting that SCR-ZIP may not be an ideal control for ZIP. These findings suggest the existence of an alternative target of ZIP that can disrupt L-LTP and memory in PKMζ-knockout mice. Consistent with this hypothesis, a recent study has reported that phosphorylation of PKCι/λ, another ZIP-sensitive atypical isoform, increases in PKMζ-knockout mice^31,33^. Inhibition of PKCι/λ by shRNA reversed L-LTP and impaired long-term memory in PKMζ-knockout mice^33^. Together, these findings raise the question of whether ZIP also targets molecules other than PKMζ.

Glycogen synthase kinase 3 (GSK-3) is a multi-functional enzyme that plays important roles in signal transduction; it regulates Wnt, protein kinase A (PKA), Hedgehog, transforming growth factor-β (TGF-β), and phosphatidylinositol 3-kinase (PI3K)-dependent insulin signaling^34–37^. Of the two GSK-3 isoforms, GSK-3β is highly expressed at neuronal synapses and is involved in synaptic plasticity^38^. GSK-3β is a constitutively active kinase and its activity is primarily regulated by the phosphorylation status of its Ser-9 residue. Phosphorylation of the Ser-9 residue inhibits GSK-3β activity, whereas dephosphorylation activates GSK-3β^34–37^.

In the present study, we performed a kinome-wide screen of candidate kinases for ZIP-dependent modulation of activity through an in vitro kinase assay. Strikingly, many kinases and phosphatases structurally independent from PKC/PKMζ were strongly inhibited at relevant concentrations, suggesting that ZIP has widespread off-target effects. In contrast, GSK-3β was activated several fold by ZIP. Intriguingly, both ZIP treatment and GSK-3β activation induce the endocytosis of GluA2-containing AMPA receptors, which may be a potential cellular substrate for memory disruption^28,39,40^. These findings have led us to propose the hypothesis that GSK-3β is a direct target of ZIP.

## Results

### ZIP directly activates GSK-3β

**Figure 1:**
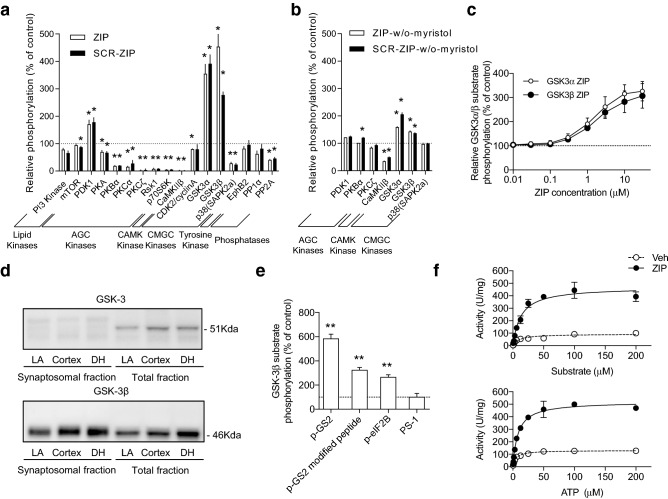
In vitro kinase assay reveals that GSK-3β is directly activated by ZIP. (**a**) Activities of various protein kinases and protein phosphatases modulated by 10 µM of myristoylated ZIP or SCR-ZIP (n = 9 for GSK-3α and β, n = 4 for PKBα, n = 2 for PKCζ, and n = 3 for others). Significance of the increase or decrease of kinase activity by ZIP and SCR-ZIP is revealed by one sample t-test. *p < 0.05 (AGK kinases: protein kinases A/G/K families, CAM kinases: calcium/calmodulin kinases, CMGC kinases: cyclin-dependent kinase (CDK), mitogen-activated protein kinase (MAPK), glycogen synthase kinase (GSK), CDC-like kinase (CLK)). The dashed line represents activities of protein kinases and protein phosphatases in the absence of the peptides. Activities of various protein kinases and protein phosphatases with ZIP or SCR-ZIP were normalized to 100%. (**b**) Activities of protein kinases and protein phosphatases modulated by 10 µM of non-myristoylated ZIP and SCR-ZIP (n = 2 per group). Significance of the increase or decrease of kinase activity by non-myristoylated ZIP and SCR-ZIP is revealed by one sample t-test. *p < 0.05 (**c**) In vitro experiments shows that ZIP activated purified GSK-3α and GSK-3β in a concentration-dependent manner (n = 3 per group). (**d**) Synaptosomal and total fractions of each brain regions (LA: lateral amygdala, Cortex: cortical brain area obtained in same coronal plane of brain slices including lateral amygdala, DH: dorsal hippocampus) were sampled and further blotted with anti-GSK-3α or β antibody. GSK-3α was not detected in the synaptosomal fraction, while GSK-3β was expressed in both the synaptosomal and total fractions. Full-length blots are displayed in Fig. S3a and b. (**e**) Activation of purified GSK-3β (12.5 nM) by ZIP (10 µM) was observed only when pre-phosphorylated substrates were used (n = 3 per group). The phosphorylation level in the presence of ZIP was normalized to that in the presence of vehicle. The concentration of GSK-3β substrate was 20 µM. One sample t-test reveals that ZIP significantly enhanced phosphorylation of prephosphorylated substrates, but not of a non-phosphorylated substrate. The prefix ‘p-’ means that the substrate is pre-phosphorylated. **p < 0.01 (**f**) ZIP facilitated the reaction kinetics of GSK-3β, which were assayed using the peptide (636–661) derived from human muscle glycogen synthase 1. Reaction curves between GSK-3β and the substrate (upper) and between GSK-3β and ATP (lower) are shown (n = 2 per group). Solid curves indicate the reaction in the presence of ZIP (10 µM), and dashed curves indicate the reaction in the presence of vehicle.

To screen for potential off-target effects induced by ZIP and scrambled ZIP (SCR-ZIP), a radiometric in vitro kinase assay was conducted with various protein kinases across the kinome. Because myristoylated ZIP was used in previous studies to make ZIP cell-permeable, we used the same myristoylated ZIP for this experiment. At concentrations used in vitro and in vivo previously, both ZIP and SCR-ZIP displayed strong modulation of several kinases, including PKCζ, PKBβ, PKCα, P70S6K, and p38, as well as phosphatases PP1α and PP2A (Fig. 1a). Notably, both GSK-3α and -3β were highly activated by ZIP (and SCR-ZIP) compared to other protein kinases. In contrast, non-myristoylated ZIP and non-myristoylated SCR-ZIP had less effect on kinase activities (Fig. 1b), suggesting that myristoylation may enhance the off-target effects induced by ZIP. This suggests that the amphiphilic structure of ZIP, which consists of a hydrophilic charged peptide and a myristoyl group, is important in the modulation of several kinases, including the original target, PKCζ. The activities of both GSK-3α and -3β were enhanced several-fold in a concentration-dependent manner (Fig. 1c). This was of substantial interest due to the previous implication of GSK-3 in synaptic depression, memory impairment, and neurodegeneration^41^.

Immunoblot analysis of brain tissue showed that in the synaptosomal fraction, GSK-3β was abundant while GSK-3α was not detected (Fig. 1d), suggesting that GSK-3β plays a larger role than GSK-3α in synaptic plasticity and memory. Therefore, we focused on the activation of GSK-3β by ZIP in subsequent experiments. It is known that many GSK-3 substrates require pre-phosphorylation at a site proximal to the GSK-3 target site, a process known as priming^34,42^. Interestingly, ZIP preferentially activated GSK-3β phosphorylation with the primed substrates (Fig. 1e). The effect of GSK-3β activation was prominent with primed glycogen synthase 2 (p-GS2) (567.4%), which has three priming phosphorylation sites, compared to modified p-GS2 (322.7%) or primed eukaryotic initiation factor 2B (p-eIF2B) (265.5%), which each have a single priming phosphorylation site. The unprimed PS-1 peptide did not show enhanced phosphorylation by GSK-3β in the presence of ZIP (115.6%). In addition, we analyzed the reaction kinetics between GSK-3β and either its substrate or adenosine triphosphate (ATP) (Fig. 1f) in the presence of ZIP. ZIP increased V_*max*_ and *K*_*m*_ for both the substrate and ATP, but the increase in V_*max*_ was much larger than that in *K*_*m*_ (V_*max*_-substrate: 93.8 U/mg increased to 469.3 U/mg; V_*max*_-ATP: 131.3 U/mg increased to 520.1 U/mg; *K*_*m*_-substrate: 10.1 μM increased to 13.1 μM; *K*_*m*_-ATP: 4.9 μM increased to 8.3 μM). These results suggest that ZIP may facilitate an enzymatic process between GSK-3β binding and phosphorylation.

### GSK-3β mediates ZIP-induced reversal of synaptic potentiation

**Figure 2:**
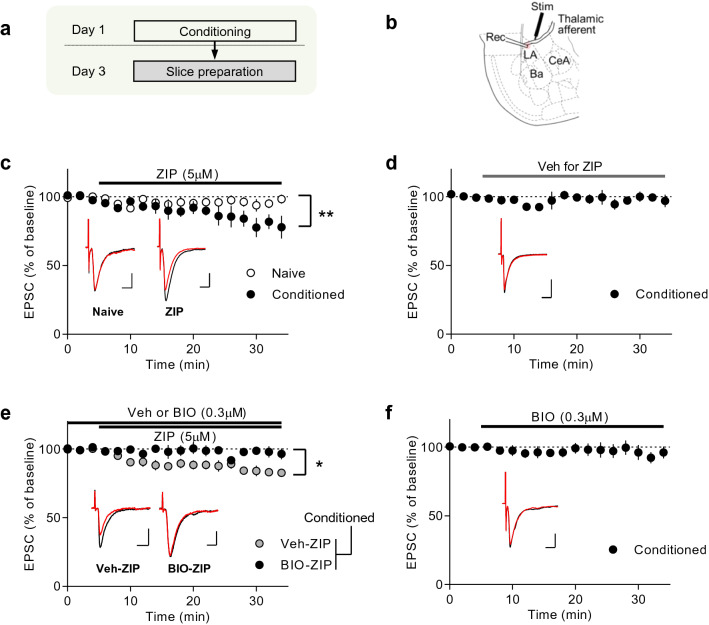
ZIP induces depotentiation of conditioning-induced potentiation at LA synapses, and BIO-acetoxime, an inhibitor of GSK-3β, blocks the ZIP-induced depotentiation. (**a**) Schematic of experimental design. Brain slices were prepared from naïve or fear-conditioned animals at 48 h after conditioning. (**b**) Schematic diagram depicting a recording site and the location of a stimulating electrode. (**c**) ZIP (5 µM) induced synaptic depression at LA synapses in brain slices from fear-conditioned rats but not from naïve rats. ZIP was applied after 5 min of baseline recording. (**d**) The vehicle (0.05% DMSO in aCSF) used for ZIP was applied after stable baseline was achieved. Application of the vehicle did not produce any significant changes in T-LA synaptic transmission in slices prepared from conditioned rats (*p* > 0.05; Wilcoxon Matched Pairs Signed Rank test vs. baseline) (**e**) BIO-acetoxime (0.3 μM) inhibited ZIP-induced depotentiation. BIO-acetoxime or vehicle was present throughout the recording. The whole-cell experiments in both groups were performed using slices prepared from fear-conditioned rats. (**f**) BIO-acetoxime had no significant effects on basal synaptic transmission at LA synapses from conditioned rats (*p* > 0.05; Wilcoxon Matched Pairs Signed Rank test vs baseline). *p < 0.05; **p < 0.01.

Depotentiation has been suggested as a mechanism of memory erasure^43–45^. Previous studies have shown that ZIP induces the reversal of LTP, a cellular substrate of memory^15,20–22,29,31^, but there is no evidence that ZIP induces reversal of learning-induced synaptic potentiation. We therefore determined whether ZIP induces depotentiation in vivo and whether blocking GSK-3β activity could attenuate the depotentiation. To this end, we chose to monitor thalamo-amygdala synapses, where synaptic efficacy is potentiated following auditory fear conditioning^45–47^. If ZIP only depotentiates synapses potentiated by fear conditioning, ZIP would depress excitatory synaptic transmission in slices prepared from conditioned animals but not from naïve animals. To test this, we prepared brain slices from naïve animals and animals that underwent fear conditioning two days before the whole-cell recordings of thalamo-amygdala synaptic transmission (Fig. 2a,b). ZIP application depressed excitatory synaptic transmission at thalamo-amygdala synapses in the slices prepared from fear-conditioned animals, but not in slices prepared from naïve animals (naïve animals, 95.2 ± 3.5%, n = 11, *p* > 0.05; conditioned animals, 79.4 ± 6.4%, n = 8, *p* < 0.05; Wilcoxon Matched Pairs Signed Rank test vs. baseline, Fig. 2c). We confirmed that the vehicle for ZIP was not responsible for the depression or depotentiation found here (99.0 ± 3.5%, n = 5, *p* > 0.05, Fig. 2d). In addition, synaptic responses after ZIP application in the conditioned group was significantly depressed compared with the naïve group (Mann–Whitney test, *p* < 0.05). This finding suggests that ZIP produces a depotentiation of conditioning-induced potentiation. ZIP-induced depotentiation was blocked by the GSK-3β inhibitor, 6-bromoindirubin-3-acetoxime (BIO-acetoxime) (vehicle, 83.4 ± 2.6%, n = 13; BIO-acetoxime, 98.0 ± 2.9%, n = 9; Mann–Whitney test, *p* < 0.01, Fig. 2e). In support, ZIP application reversed pre-established late-phase long-term potentiation, and this reversal was also blocked by BIO-acetoxime (see Supplementary Fig. S1). Application of BIO-acetoxime alone had no effect on excitatory synapses at the thalamo-amygdala synapses in the slices prepared from fear-conditioned animals (94.6 ± 4.4%, n = 8, *p* > 0.05; Wilcoxon Matched Pairs Signed Rank test vs. baseline, Fig. 2f). These data suggest that GSK-3β activity is required for ZIP-induced depotentiation of conditioning-induced potentiation at thalamo-amygdala synapses.

### GSK-3β is required for ZIP-induced memory disruption

**Figure 3:**
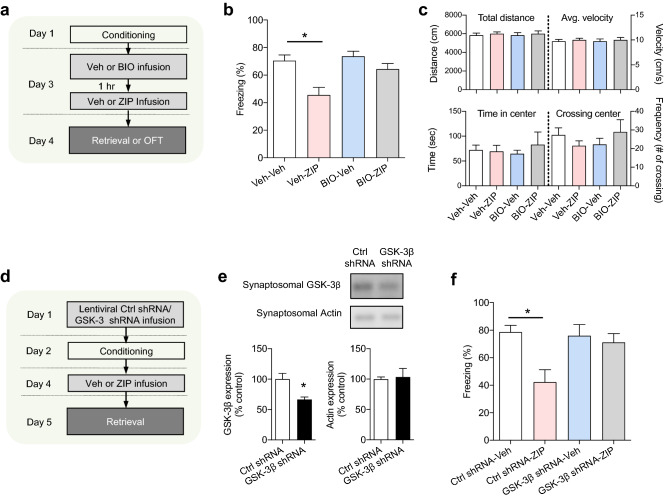
Pre-injection of BIO-acetoxime or GSK-3β shRNA attenuates ZIP-induced memory disruption. (**a**) Schematic of experimental design for experiments shown in (**a**)–(**c**). Rats were fear-conditioned on Day 1. BIO-acetoxime (or vehicle) was microinjected 1 h before the ZIP (or vehicle) injection on Day 3. ZIP (10 nmol/µL/hemisphere) or BIO-acetoxime (75 pmol/0.5 µL/hemisphere) was injected into the LA. The memory retention test was performed on Day 4. Schematic illustrations of injection target are displayed in Fig. S2a and b (**b**) Pre-injection of BIO-acetoxime attenuated ZIP-induced memory disruption. Veh-Veh (vehicle of BIO-acetoxime for 1st injection, vehicle of ZIP for 2nd injection), Veh-ZIP (vehicle of BIO-acetoxime for 1st injection, ZIP for 2nd injection), Bio-Veh (BIO-acetoxime for 1st injection, vehicle of ZIP for 2nd injection), Bio-ZIP (BIO-acetoxime for 1st injection, ZIP for 2nd injection). (**c**) Open field test was performed for all experimental groups. A new set of rats were used for the open field test. No significant difference was observed between the groups. (**d**) Schematic of experimental design for experiments shown in (**d**)–(**f**). Lentiviral particles containing GSK-3β shRNA or control shRNA were injected into the LA 24 h before fear conditioning. Vehicle or ZIP was injected 48 h after fear conditioning, and the memory retention test or the preparation of LA synaptosomes was performed on the following day. Schematic illustrations of injection target are displayed in Fig. S2c. (**e**) Synaptosomal GSK-3β (left) but not synaptosomal actin (right) was reduced by microinjection of GSK-3β shRNA. Full-length blots are displayed in Fig. S3c–f. (**F**) Pre-injection of GSK-3β shRNA attenuated ZIP-induced memory disruption. Ctrl shRNA-Veh (Ctrl shRNA for 1st injection, vehicle of ZIP for 2nd injection), Ctrl shRNA-ZIP (Ctrl shRNA for 1st injection, ZIP for 2nd injection), GSK-3β shRNA-Veh (GSK-3β shRNA for 1st injection, vehicle of ZIP for 2nd injection), GSK-3β shRNA-ZIP (GSK-3β shRNA for 1st injection, ZIP for 2nd injection). *p < 0.05.

Next, we determined whether GSK-3β activation would also be required for ZIP-induced memory disruption. Animals underwent fear conditioning, and two days later BIO-acetoxime (or vehicle) was microinjected into the LA 1 h before ZIP (or vehicle) injection. Then, freezing responses to the conditioned stimulus were measured 24 h after the ZIP (or vehicle) injection (Fig. 3a). Consistent with previous studies, ZIP injection following vehicle injection (Veh-ZIP group) reduced fear responses compared to the vehicle -vehicle group (in which the vehicle was injected following the vehicle injection) (Veh-ZIP, 45.63 ± 5.46%, n = 21; Veh-Veh, 70.53 ± 4.07%, n = 14; *p* < 0.001, H = 17.04, Kruskal–Wallis test; *p* < 0.05, post-hoc Dunn’s multiple comparison) (Fig. 3b). In contrast, ZIP injection following BIO-acetoxime injection (BIO-acetoxime-ZIP group) did not affect freezing responses compared to the BIO-acetoxime-Veh group (in which the vehicle was injected following BIO-acetoxime injection) (BIO-acetoxime-ZIP, 64.37 ± 4.03%, n = 26; BIO-acetoxime-Veh, 73.73 ± 3.59%, n = 16; *p* > 0.05, post-hoc Dunn’s multiple comparison). The differences between the groups were not due to non-specific increases in anxiety levels or locomotive activities by drug injections because behaviors in an open field were indistinguishable between the four groups (time in center, Veh-Veh, 72.2 ± 9.9 s, n = 8; Veh-ZIP, 69.2 ± 12.5 s ,n = 7; BIO-Veh, 64.7 ± 7.0 s, n = 7; BIO-ZIP, 82.9 ± 25.6 s, n = 7, total distance Veh-Veh, 5841.8 ± 226.7 cm, n = 8; Veh-ZIP, 6013.9 ± 190.2 cm, n = 7; BIO-Veh, 5848.0 ± 275.0 cm, n = 7; BIO-ZIP, 6010.0 ± 289.1 cm, n = 7; Fig. 3c). These findings suggest that ZIP induces memory disruption via GSK-3β activation. In order to achieve more specific inhibition of GSK-3β, we chose to use GSK-3β shRNA. We knocked down GSK-3β by injecting GSK-3β shRNA one day prior to initial conditioning (Fig. 3d). ZIP was injected two days after the conditioning and freezing responses to the conditioned stimulus was tested on the following day. As a control, control (scrambled, blank targeting) shRNA or vehicle was injected in place of GSK-3β shRNA or ZIP, respectively. We first determined whether microinjection of GSK-3β shRNA would reduce the expression of synaptic GSK-3β. GSK-3β shRNA was injected into the LA one day prior to conditioning and brain slices were prepared when ZIP was supposed to be microinjected. The level of synaptosomal GSK-3β expression in the lateral amygdala was significantly reduced by the injection of GSK-3β shRNA (n = 5 from 15 rats, *p* < 0.05, Kruskal–Wallis test, Fig. 3e). Having established that GSK-3β shRNA was effective under our conditions, we determined whether knockdown using GSK-3β shRNA would attenuate the amnesic effects of ZIP. Indeed, ZIP injection failed to induce an amnesic effect on conditioned memory when GSK-3β shRNA was pre-injected (shRNA-ZIP, 71.13 ± 6.42%, n = 10; shRNA-Veh, 75.98 ± 8.12%, n = 8; *p* < 0.01, H = 11.68, Kruskal–Wallis test; *p* > 0.05, post-hoc Dunn’s multiple comparison, Fig. 3f). In contrast, ZIP injection still produced an amnesic effect on conditioned memories when control shRNA was pre-injected (Ctrl shRNA-ZIP, 42.32 ± 8.98%, n = 11; Ctrl shRNA-Veh, 78.61 ± 4.90%, n = 10; *p* < 0.05, post-hoc Dunn’s multiple comparison, Fig. 3f). Taken together, these results indicate that GSK-3β is required for ZIP-induced memory disruption of conditioned fear.

### Activation of GSK-3β is sufficient to produce memory disruption

**Figure 4:**
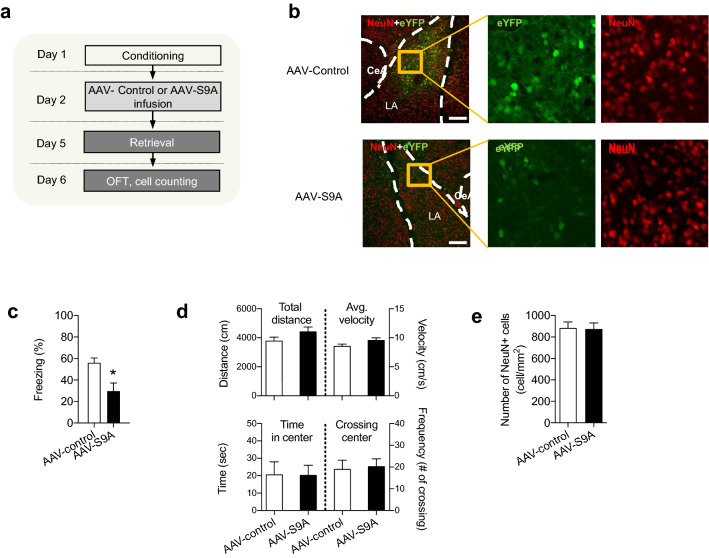
Expression of a non-inhibitable form of GSK-3β induces memory disruption. (**a**) Schematic of experimental design. AAV-control (n = 7) or AAV-S9A (n = 7) was microinjected into the LA 24 h after conditioning. The memory retention test was preformed 72 h after the injection. The open field test was then performed 24 h after the retention test. Lastly, the subjects were sacrificed to count viable cells in the LA. (**b**) Confocal images were obtained for cell counting on Day 6. Low-magnification images (left) showed both NeuN immunoreactivity and eYFP expression in the two groups (AAV-control and AAV- S9A). Images on the right show the designated areas in the left images magnified, showing eYFP expression and NeuN immunoreactivity. The number of NeuN immunoreactive cells was not different between the two groups, whereas the number of eYFP fluorescence-labeled cells was lower with AAV- S9A than with AAV-control. (**c**) Microinjection of AAV-S9A reduced freezing responses to the CS (i.e., fear memory retrieval) on Day 5 compared with the AAV-control. The scale bar indicates 200 µm. *p < 0.05. (**d**) Open field test was performed for the AAV-control and AAV-S9A groups on Day 6. No significant difference was observed between the groups. (**e**) NeuN immunoreactivity was assessed using the rats undergoing the open field test on Day 6. The number of NeuN-positive cells in the LA was not different between the two groups.

One obvious question is whether GSK-3β activation alone is sufficient for inducing memory disruption. This would be the case if ZIP induces memory disruption solely via GSK-3β activation. To test this, we determined whether exogenous expression of GSK-3β is sufficient for memory disruption of learned fear. Since GSK-3β is inhibited powerfully via phosphorylation at Ser 9, we used a GSK-3β mutant in which the Ser9 was replaced with alanine. At the same time, we ensured that the expression of the S9A mutant GSK-3β was as weak and transient as possible in order to avoid possible side effects caused by overexpression. When the viral vector carrying the S9A gene was injected into the LA, the minimum time for expression of the S9A mutant appeared to be three days (Fig. 4a). Indeed, we could not detect GSK-3β proteins within two days after the injection. We first tested whether the S9A mutant was expressed adequately. Since the S9A gene was conjugated to the eYFP gene, which meant that the S9A mutant proteins were cleaved post-translationally from eYFP proteins, we counted the number of eYFP-expressing cells three days after injection to measure the expression of S9A mutant proteins (see the Methods section for additional information). The S9A mutant gene appeared to be expressed in 17.10 ± 2.33% of NeuN positive cells (see Fig. 4b). The low percentage of cells expressing eYFP suggests that the expression level of the GSK-3β mutant was very weak. Microinjection of viral vectors (adeno-associated virus, AAV) carrying the S9A mutant gene produced memory disruption of learned fear relative to control vectors carrying the eYFP gene when freezing was tested three days after the injection (AAV-S9A, 29.8 ± 7.5%, n = 7; AAV-control, 56.1 ± 4.5%, n = 7; *p* < 0.05, Fig. 4c). The differences between the groups were not due to non-specific increases in anxiety levels or locomotive activities by drug injections, because behaviors in an open field were indistinguishable between the two groups (time in center, AAV-S9A, 20.3 ± 3.5 s, n = 7; AAV-control, 19.1 ± 4.0 s, n = 7; total distance AAV-S9A, 4442.2 ± 300.8 cm, n = 7; AAV-control, 3803.5 ± 245.2 cm, n = 7; *p* < 0.05, Fig. 4d) In addition, the microinjection of viral vectors carrying the S9A mutant gene did not affect the number of cells in the LA compared to the control vectors, arguing against the possibility that the expression of S9A mutant induces neuronal cell death (AAV-S9A, 874.1 ± 56.2 cells/mm^2^, n = 7; AAV-control, 887.6 ± 56.8 cells/mm^2^, n = 7, *p* = 0.78; Fig. 4e). Together, these findings suggest that GSK-3β activation alone may be sufficient for memory disruption of learned fear.

## Discussion

Our findings suggest that activated GSK-3β is a key off-target effector of ZIP and contributes to ZIP-induced memory disruption. In vitro kinase assays showed that although various protein kinases and protein phosphatases were modulated by ZIP and SCR-ZIP, GSK-3β was the most robustly activated by ZIP, and the effects of ZIP on GSK-3β depended on the myristoyl group of ZIP. Consistent with the effects of ZIP on LTP^21^, application of ZIP induced ex vivo depotentiation of conditioning-induced potentiation at thalamo-amygdala synapses (see ref.^46–48^). The ZIP-induced depotentiation was blocked by BIO-acetoxime, a potent and selective GSK-3β inhibitor. In addition, ZIP-induced memory disruption of auditory-fear memory was also blocked by the GSK-3β inhibitor and GSK-3β-targeted shRNA in the LA. Lastly, conditioned fear was decreased by the expression of a non-inhibitable form of GSK-3β in the LA.

Parsons and Davis^49^ have suggested that the changes in behavior (e.g., fear startle) following ZIP administration reflect changes in behavioral performance rather than memory. This proposal is based on the evidence that the effects of ZIP on learned fear behavior are not permanent. However, there have been debates regarding this subject and some researchers argue against the proposal^28,32,50,51^ (see also ref.^52^). As suggested by Parsons and Davis^49,52^, one way to confirm the amnesic effect of ZIP would be to test memory for over a week after ZIP is injected; In fact, the two previous studies met this condition^24,32^. It remains to be elucidated whether ZIP induces permanent amnesia in diverse forms of memory. In the present study, we provide evidence that conditioning-induced potentiation at LA synapses, a fear memory trace^46–48^, is reversed by ZIP (Fig. 2). This further implies that ZIP impairs memory storage itself within the timescale tested. Thus, our findings support the possibility that ZIP alters memory storage.

In the study by Chew et al.^53^, GSK-3β was knocked down using lentiviral shRNA that were injected into the dentate gyrus around one month before the LTP and fear conditioning experiments were conducted. They have found that the GSK-3β knockdown impairs contextual fear conditioning but not cued fear conditioning. The findings in this previous study seem to be inconsistent with our data; that is, our present findings suggest that lowered activity of GSK-3β may enhance memory expression. These authors have found that retrieval of contextual fear memory decreases in the GSK-3β-knockdown group. The inconsistency could be due to many reasons. First, compensatory changes after GSK-3β knockdown may account for the knockdown effect on contextual fear memory. Second, GSK-3β knockdown may affect other stages of memory formation such as memory acquisition and consolidation; thus, GSK-3β knockdown after memory consolidation might produce different effects on contextual fear memory. Intriguingly, LTP was boosted in the GSK-3β knockdown group, which is consistent with our findings. Therefore, more careful studies are needed to clarify the issues surrounding GSK-3β and memory disruption in the future.

Previous studies have reported that ZIP has toxic effects on cultured neurons^54^, and transient silencing effects on local field potentials in vivo^55^. In the present study, we found that ZIP injection into the LA induced no significant effects on the LA field potential and whole-cell currents in slices prepared from naïve rats (see Fig. 2 and S1). Furthermore, ZIP injection into the LA did not change anxiety levels (Fig. 3c). This is consistent with the fact that the LA is not involved in anxiety^56^. No significant changes in anxiety in the ZIP-injected group also suggest that ZIP, which is injected into the LA, does not damage the central amygdala, which is critical for anxiety^56^. In the present study, we used the same amount of ZIP as in previous behavioral studies^57^, which had reported that cell viability is not affected 24 h after ZIP injection. Thus, the ZIP effects observed in the present study are unlikely to be caused by toxicity or silencing.

Although ZIP has been used as an inhibitor of PKC/PKMζ, the specificity of its effects is controversial^29,58–61^. Consistently, our study has demonstrated ZIP affects a diverse range of protein kinases (Fig. 1a). Kinases spanning the AGC, CAMK, CGGC kinase groups as well as several protein phosphatases were inhibited, whereas GSK-3α and β were activated. It is possible that ZIP, by chance, acts on an optimal set of multiple targets that are sufficient for triggering memory erasure. Our in vitro experiments suggest that ZIP-induced activation of GSK-3β has at least some specificity; that is, ZIP activates GSK-3β activity by enhancing the interaction between GSK-3β and ATP (see Fig. 1f), and that ZIP-induced activation of GSK-3β prefers highly primed substrates (see Fig. 1e). As shown in Fig. 1., the in vitro experiment has shown that SCR-ZIP affects numerous kinases in a way similar to ZIP. Although some of previous studies have shown that SCR-ZIP has much less effects on PKMζ activity in vitro^15,20,59^, other studies have demonstrated that SCR-ZIP also has a significant inhibitory effect on PKMζ^29,30,54^. In one study^29^ showing the effects of SCR-ZIP, the authors have tested whether myristoylation is necessary for its effect. However, another myristoylated peptide (PKI) had no effects on L-LTP in this particular study, suggesting that myristoylation alone cannot account for the ZIP effect. Consistent with studies showing comparable effects between ZIP and SCR-ZIP, we have also observed significant effects of SCR-ZIP, which is myristoylated, on GSK-3β activity. Furthermore, non-myristoylated ZIP and SCR-ZIP peptide show much less effects on various kinases (see Fig. 1b). Although we have not tested other types of myrisotylated peptides, it is likely that such peptides would have no significant effects on GSK-3β. Taken together, we have hypothesized that along with myristoylation, the composition of amino acids in ZIP or SCR-ZIP may be critical for exerting its effects on either PKMζ or GSK-3β since the two peptides have the same amino acid composition. It will be interesting to test this possibility in the future. Furthermore, it is intriguing that several known activators of GSK-3β such as sulfatide (3-O-sulfogalactosylceramide), phosphatidylinositol, and phosphatidylserine^62^ as well as ZIP (myristoylated-ZIP) all possess amphiphilic structures with long hydrophobic tails. This suggests that the structural motif shared by these molecules affords a key to endogenous and exogenous GSK-3β activation and may provide avenue for further investigating the specific role of GSK-3β in the brain.

Previous research has demonstrated that GSK-3β activation is required in LTD, while LTP inhibits GSK-3β^38,63,64^. Here, we demonstrate a novel role of GSK-3β in ZIP-mediated depotentiation. This is consistent with the previous data showing that the inhibition of GSK-3 upstream kinases, which enhances GSK-3β activity, blocks LTP or induces depotentiation of L-LTP^64–67^ In addition, it has been reported that GSK-3β is involved in memory formation. Overexpression of GSK-3β impairs hippocampus-dependent spatial memory formation in the Morris water maze^68^. Enhancements in GSK-3β activity by inhibiting GSK-3 upstream kinases such as PI3 kinase and PKC impair spatial memory formation^69^. Therefore, our data with the previous findings support the possibility that GSK-3β plays a pivotal role in regulating memory maintenance.

GSK-3β activation is known to be required for LTD at naïve synapses^63^, as supported by findings that GSK-3β inhibitors block LTD induction. However, this does not guarantee that the activation of GSK-3β is sufficient to induce LTD. Our results in Fig. 2c have shown that ZIP, which is an activator of GSK-3β, failed to induce LTD at naïve synapses. This may suggest that GSK-3β activation alone is insufficient to induce LTD. Instead, our present findings indicate that ZIP alone is sufficient to induce depotentiation. As shown in Fig. 2, ZIP-induced depotentiation was inhibited by the GSK-3β inhibitor, BIO-acetoxime. However, this does not preclude the possibility that other signaling molecules (including those shown in Fig. 1) are required for ZIP-induced depotentiation. Here we have found that the expression of the constitutively active form of GSK-3β alone produces memory disruption (Fig. 4), implying that GSK-3β activation on its own is sufficient to induce depotentiation. It will be interesting to determine whether the expression of constitutively active GSK-3β induces depotentiation at amygdala synapses. In support of the differential effects of ZIP on naïve and potentiated synapses, depotentiation and long-term depression have been shown to involve different molecular mechanisms^70^. It is speculated that a subset of GSK-3β substrates may be pre-primed at memory-storing synapses, which enables ZIP to have a preferential effect on these synapses (see Fig. 1f).

Our findings suggest that GSK-3β activity should be regulated tightly within a limited range to prevent deleterious memory loss. Otherwise, memory maintenance would be defective, especially in pathological conditions where GSK-3β is overactivated. Indeed, both GSK-3β overactivation and memory defects are observed in neurological disorders such as Alzheimer’s disease, Parkinson’s disease, major depression, bipolar disorders, and schizophrenia^71–75^. GSK-3β may have an additional active role in via synaptic depotentiation in normal brains. In support of this, endogenous amyloid beta, which can reduce synaptic strength via GSK-3β activation, is required for memory forgetting in normal brains^76^.

Based on the present findings, we propose that ZIP directly activates GSK-3β, leading to a depotentiation at memory storing synapses. Since ZIP induces catastrophic and irreversible amnesia, it is likely that GSK-3β activation induces a persistent change of memory-storing synapses (i.e., changes in synaptic structures, synaptic loss etc.). Consistently, GSK-3β activation is implicated in PSD-95 mobilization^77^ or reversible synaptic loss^78^. ZIP-induced endocytosis of GluA2-containing AMPA receptors (see ref.^28,63^) may be a molecular mechanism for the potential structural changes induced by ZIP, since GluA2 on the membrane acts as a critical scaffold to support memory-storing synapses^79–81^. In the future, the development of novel GSK-3β activators may be useful to selectively erase emotional and implicit components of traumatic memories if they can be delivered or activated in the relevant area of the brain.

## Methods

### In vitro GSK-3β assay

GSK-3β experiments were performed by Millipore (KinaseProfiler and IC50) according to their KinaseProfiler protocol v58. Kinases were diluted in the appropriate buffer prior to addition to the reaction mix. ZIP was prepared to 50 × the final assay concentration in 100% DMSO. Positive control wells contained all reaction components except the compound of interest; DMSO (at a final concentration of 2%) was included in these wells to control for solvent effects. The blank wells contained all reaction components with a reference inhibitor in place of ZIP. This abolished kinase activity and established the baseline (0% kinase activity remaining). The reaction was initiated by the addition of MgATP mix. After incubation, the reaction was stopped by the addition of 3% phosphoric acid solution. 10 µL of the reaction mixture was then spotted onto a P30 filtermat and washed three times in 75 mM phosphoric acid and once in methanol for 5 min each prior to drying and scintillation counting.

Detailed information is available on their website (www.merckmillipore.com). Analysis of ZIP reaction kinetics was performed by Eurofins Pharma Discovery Services (FlexLab). Detailed information is available on their website (www.eurofins.com/discoveryservices).

### ZIP and SCR-ZIP

We obtained myristoylated ZIP (Myr-SIYRRGARRWRKL) and SCR- ZIP which consists of same amino acids as ZIP but in scrambled order (SCR-ZIP; Myr-RLYRKRIWRSAGR, cat. #0.3215) from Tocris (Bristol, UK). Non-myristorylated ZIP and SCR-ZIP were obtained from Peptron (Daejeon, South Korea).

### Animals

All procedures were approved by the Institute of Laboratory Animal Resources of Seoul National University (SNU-120330-1-1). Male Sprague–Dawley rats (3–5 weeks old) were maintained with free access to food and water and group housed (2–3 rats per cage) under an inverted 12/12-h light/dark cycle (lights off at 09:00). Animals were housed alone when they got surgical treatments to keep the wounds protected from their cage mates.

### Behavioral procedures

Behavioral training was done during the dark portion of the light/dark cycle. A rectangular Plexiglass box with a metal grid floor connected to an electrical current source (Coulbourn Instruments, Allentown, PA) was used for the conditioning context. The conditioning context was illuminated with white light and cleaned with a 70% ethanol solution. The test context used for tone test of animal was a cylindrical Plexiglass chamber with a metal grid or a flat Formica floor and was cleaned with 1% acetic acid. For fear conditioning, rats were placed in the conditioning context and left undisturbed for 2 min. A neutral tone (30 s, 2.8 kHz, 85 dB) co-terminating with an electrical foot shock (0.7 mA, 1 s) was then presented three times at an average interval of 100 s (Day 1 or 2). During the memory retrieval test (Day 4 or 5), a single CS was given to the animal after 4 min of exploration in the test context. Conditioned freezing, which was defined as immobility except for respiratory movements, was quantified by trained observers. The total freezing time during a test period was normalized against the duration of the tone (30 s). To test the general activity and anxiety of the rats, an open field test was performed. Rats were placed in an open field that consists of squares dividing into peripheral (within 20 cm from the walls) and central (40 × 40 cm) zones for 10 min. The total distance moved in the arena and the time spent in the central zone were measured using either EthoVision 3.1 software (for experiments depicted in Fig. 3; EthoVision 3.1, Noldus Information Technology, Wageningen, Netherlands) or Ethowatcher^82^ (for experiments depicted in Fig. 4). To avoid possible bias, all experiments were performed in a blinded fashion.

### Slice preparation

Brain slices were prepared using previously described protocols^45,83^. Rats were anesthetized with isoflurane and decapitated to extract the brain. Isolated whole brains were placed in an ice-cold modified artificial cerebrospinal fluid (aCSF) solution containing the following (in mM): 175 sucrose, 20 NaCl, 3.5 KCl, 1.25 NaH_2_PO_4_, 26 NaHCO_3_, 1.3 MgCl_2_, and 11 D-(+)-glucose. Solutions were then bubbled with 95% O_2_/5% CO_2_. Coronal slices (300 μm) including the LA were cut using a vibroslicer (VT1200, Leica, Nußloch, Germany) and incubated in normal aCSF containing the following (in mM): 120 NaCl, 3.5 KCl, 1.25 NaH_2_PO_4_, 26 NaHCO_3_, 1.3 MgCl_2_, 2 CaCl_2_, and 11 D-(+)-glucose, bubbled at room temperature with 95% O_2_/5% CO_2_. Immediately before transferring a slice to the recording chamber, the cortex overlying the LA was cut away with a scalpel so that cortical epileptic burst discharges would not invade the LA in the presence of picrotoxin.

### Afferent stimulation

Brain slices were selected based on the presence of a well-isolated, sharply defined trunk (containing thalamic afferents) crossing the dorsolateral division of the LA and the presence of the external capsule (containing cortical afferents)^45,84^. A concentric bipolar electrode (CBAEC75, FHC Inc., Bowdoin, ME, USA) was used for the stimulation of thalamic and cortical afferents. For the stimulation of thalamic afferents, a concentric bipolar electrode was placed at the midpoint of the trunk between the internal capsules^45^. Regions and cells of interest for all recordings were located beneath the midpoint of the trunk spanning the LA horizontally.

### Whole-cell patch-clamp recordings

Whole-cell recordings were made using an Axopatch 200A, 700A, or 700B amplifier (Molecular Devices, San Jose, CA, USA) as previously described^45^. Recordings were obtained using pipettes with a resistance of 3–4.5 MΩ. Recordings were made under infrared differential interference contrast (IR-DIC)-enhanced visual guidance from neurons that were three to four cell layers below the surface of 300-µm-thick slices at 32.0 ± 1.0 °C. The aCSF containing picrotoxin (100 µM, Sigma-Aldrich, St. Lois, MO, USA) was delivered to slices via superfusion driven by a peristaltic pump (REGLO digital Ismatec, Wertheim, Germany) at a flow rate of 1.5 mL/min. The pipette series resistance was monitored throughout the experiments. If the series resistance changed by > 20%, the data were discarded. Whole-cell currents were filtered at 1 kHz, digitized at up to 20 kHz, and stored on a microcomputer (Clampex 9 or 10 software; Molecular Devices). All recordings were completed within 4.5 h after slice preparation. Cells were classified as principal neurons based on the pyramidal shape of their somata. A minor portion (< 5%) of recorded neurons exhibited spontaneous excitatory postsynaptic currents (EPSCs) with a faster decay time and larger amplitude (> 100 pA), which are typical characteristics of interneurons in the LA, and were excluded from analysis. For the whole cell experiments, ZIP and BIO-acetoxime were dissolved in 100% DMSO before diluting with aCSF solution (1:2000). Hence, aCSF with 0.05% DMSO was the vehicle solution used for ZIP and BIO-acetoxime.

### Expression of GSK-3α and -3β in synaptosomal and total fractions

In order to determine GSK-3α and -3β expression in synaptosomal and total fractions, we obtained 400 µm-thick coronal brain slices containing the LA, the sensory cortex, or the dorsal hippocampus. Each region of the slice was cut, while ensuring the size of each is similar, and pooled (three to six pieces per rat and three rats per vial). The samples were lysed in ice-cold homogenization buffer containing 10 mM Tris (pH 7.6), 320 mM sucrose, 5 mM NaF, 1 mM Na_3_VO_4_, 1 mM EDTA, and 1 mM EGTA. The lysates were centrifuged at 1,000 × g for 10 min at 4 °C to remove nuclei and large debris. A portion of the supernatant was spared for measuring whole cell protein and the remainders were centrifuged at 10,000 × g for 30 min at 4 °C to obtain a crude synaptosomal fraction, which was then lysed in modified RIPA buffer containing 50 mM Tris (pH 7.6), 150 mM NaCl, 5 mM NaF, 1 mM Na_3_VO_4_, 0.5% Triton X-100 (vol/vol), 0.5% sodium deoxycholate (wt/vol), 0.1% SDS (wt/vol), 1 mM phenylmethylsulfonyl fluoride, 100 µg/mL aprotinin, and 100 µg/mL leupeptin. Samples were sonicated and spun down at 15,000 g at 4 °C for 15 min. Each sample was subsequently analyzed by immunoblotting with a rabbit polyclonal antibody to GSK-3α (1:1000, sc-7879, Santa-Cruz) and mouse monoclonal antibody to GSK-3β (1:1000, 610,202, BD Transduction Laboratory). The immunoblot was probed with a horseradish peroxidase-conjugated secondary antibody (goat antibody to rabbit, 1:1000, sc-2004, Santa-Cruz; and goat antibody to mouse IgG: 1:1000, sc-2005, Santa-Cruz) for 1 h and developed using an ECL-based immunoblotting detection system (Bio-Rad, Hercules, CA, USA).

### Virus preparation

Lentiviruses used for knocking down GSK-3β and its control were obtained from Santa-Cruz (GSK-3β shRNA lentivirus: sc-270460-V; control shRNA lentivirus: sc-108080), and Adeno-associated viruses used for knocking in of phosphorylation-resistant form (S9A) of GSK-3β were generously provided by Dr. Yoon et al.^85^. The viruses were produced by using AAV Helper-Free System (Agilent Technologies, Santa Clara, CA, USA) according to the manufacturer's recommended protocol. Briefly, pAAV expression vector, pAAV-RC (DJ) and pHelper were transfected into AAV-293 cells. They were further incubated for 66–72 h. Then cells were harvested and went through repeated freeze/thaw cycles. The debris were centrifuged and the supernatant was mixed with 40% (vol/vol) PEG (to final 10% PEG) and left at 4 °C for 36–48 h. The viral particles were obtained from further centrifugation and resuspension in ice-cold PBS for two times. The GSK-3β S9A [MSGRPRTTAFAES…] containing plasmid was constructed from an original vector pAAV-ef1a-DIO-eYFP (from Karl Deisseroth, addgene #27,056, Water Town, MA, USA). The final form is pAAV-ef1a-S9A-P2A-eYFP where P2A is a linker peptide that is cleaved post-translationally, and the cleavage produces functional S9A and eYFP proteins. The viral titer (~ 1.0 × 10^8^ infectious unit/µl) was measured in HT-1080 cells via flow cytometry.

### Cannula implantation and virus/drug infusion

Rats were anesthetized with an intraperitoneal injection of pentobarbital sodium (50 mg/kg), mounted on a stereotaxic apparatus (Stoelting, Wood Dale, IL, USA), and 26-gauge stainless-steel cannulas (model C315G, Plastics One Inc., Roanoke, VA, USA) were implanted bilaterally into the LA (anterior–posterior: − 2.35 mm, medial–lateral: ± 5.05 mm, and dorsal–ventral: − 6.7 mm) using previously described techniques^45,86^. A 32-gauge dummy cannula was inserted into each cannula to prevent clogging. Two jewelry screws were implanted over the skull to serve as anchors, and the whole assembly was affixed to the skull with dental cement. Rats were given at least 1 week to recover before experiments were performed. Following completion of the experiments, the intra-LA placement of the injection cannula tips was confirmed. Briefly, rats were anesthetized with urethane (1 g/kg of body weight, intraperitoneally) and transcardially perfused with 0.9% saline solution (wt/vol) followed by 10% buffered formalin (vol/vol). Brains were extracted and post-fixed overnight. Coronal Sects. (80 μm thick) were cut using a vibroslicer (NVSL, World Precision Instruments, Sarasota, FL, USA), stained with cresyl violet, and examined under a light microscope. BIO-acetoxime (75 pmol/0.5 µl/hemisphere) and ZIP (10 nmole/µl/hemisphere) were dissolved in 50% DSMO/Tris saline and administered bilaterally into the LA via 33-gauge injector cannulas (C315I, Plastics One Inc.) attached to a 10 μl Hamilton syringe at a rate of 0.2 μl/min. The concentration of BIO-acetoxime for microinjection was determined based on a previous study^87^ and the 1 h interval between BIO-acetoxime and ZIP injections was determined based on a previous study^86^. The delivery of control-shRNA/GSK-3β-shRNA lentiviral particles and control AAV-eYFP/AAV-S9A-eYFP were conducted in a similar way (2 µl/hemisphere for lentiviral particles, 1 µl/hemisphere for AAVs). Following infusion, cannulas were left in place for an additional minute to allow for diffusion of the drugs or the vectors. The injector cannulas were then replaced with dummy cannulas, and the rats were returned to their home cage. For the AVV experiments, we included the rats in which eYFP expression was confined within both sides of the LA.

### Quantification of synaptosomal GSK-3β in the LA after viral injection

Microdissected LA areas from 400-µm-thick brain slices were pooled (three to six pieces per rat and three rats per vial), and synaptosomal proteins were obtained as described earlier. The total protein density of lysed synaptosomal proteins was measured via BSA assay. 4.33 µg of each sample was subsequently analyzed by immunoblotting with a monoclonal antibody to GSK-3β (1:1000, 610,202, BD Transduction Laboratory) and polyclonal antibody to Actin (1:2000, sc-1616, Santa-Cruz). The immunoblot was probed with a horseradish peroxidase-conjugated secondary antibody (goat antibody to mouse IgG: 1:1000, sc-2005; and donkey antibody to goat IgG, sc-2020, 1:5000; Santa-Cruz) for 1 h and developed using an ECL-based immunoblotting detection system (Bio-Rad). The relative optical densities of the bands were quantified using luminograph II image analysis software (Atto Technology, Amherst, NY, USA). The linearity of the immunoblotting results was confirmed by analyzing the relative optical band densities of serially diluted samples (pooled from the used samples) loaded on each gel. Optical densities of the bands were normalized with respect to those of the control-shRNA injected group in each experiment.

### Immunohistochemistry and NeuN + cell counting

Immediately after the open field test, the rats were anesthetized with urethane and transcardially perfused to reveal whether neuronal viability is affected by each virus. The brains were extracted and post-fixed overnight. The amygdala-containing sections (60 μm thick) were obtained from a region 2.0–3.0 posterior to bregma using a vibroslicer (NVSL; World Precision Instruments) and stored in PBS. The sections were incubated in 1% sodium borohydride for 30 min, washed 3 times in PBS and pre-incubated in a blocking solution (10% goat serum, 1% BSA, 0.3% Triton-X100 in PBS). Then, sections were incubated in a primary antibody solution containing NeuN antibody (Millipore, 1:2000) in 1% goat serum, 1% BSA, and 0.3% Triton-X100 in PBS for 1 h, followed by incubation in the fluorescent secondary antibodies (Merck, 1:500) for 2 h. Cell counting was conducted in a similar way as previously described^88,89^ with slight modifications. The sections were examined under confocal microscopy (LSM 710 microscope, Zeiss, Oberkochen, Germany). The area inside of external and internal capsule within 1200 µm from the lateral tip is considered as LA. In a 1-in-4 series of sections, the regions of interest (ROI) were sampled in unbiased stereoscopic approach (counting frame size: 49 × 49 μm, total 4% of image area is sampled), and NeuN-positive cells in the ROI areas were counted. The optical dissector height was 10 μm.

### Statistical analysis

Sample size was determined on the basis of those used in our previous studies^45,84,90^. Subjects were randomly assigned to the experimental groups by a person who was not directly involved in the experiments. Comparisons of data among three or more groups were made using a one-way ANOVA on ranks (Kruskal–Wallis test) with a subsequent Dunn’s multiple comparison test. Comparisons between two groups were made using the Mann–Whitney test. The kinase assay results shown in Fig. 1 were analyzed using a one-sample t-test. In the Fig. 2 experiments, we averaged the EPSCs (% of baseline) during the last 5 min of each recording as previously described^45,84,90^ ; afterwards, we used the Wilcoxon Matched Pairs Signed Rank test and the Mann–Whitney test for within-group and between-group comparions, respectively. A *p* value < 0.05 was considered indicative of statistical significance. All values except those in Fig. 1f (mean ± SD) are expressed as mean ± SEM.

## Supplementary information

Supplementary Information.

## References

[1] Bliss TVP, Collingridge GL (1993). A synaptic model of memory: long-term potentiation in the hippocampus. Nature.

[2] Roberson ED (1999). The mitogen-activated protein kinase cascade couples PKA and PKC to cAMP response element binding protein phosphorylation in area CA1 of hippocampus. J. Neurosci..

[3] Schwartz JH, Greenberg SM (1987). Molecular mechanisms for memory: second-messenger induced modifications of protein kinases in nerve cells. Annu. Rev. Neurosci..

[4] Malinow R, Schulman H, Tsien RW (1989). Inhibition of postsynaptic PKC or CaMKII blocks induction but not expression of LTP. Science (80-. )..

[5] Blitzer RD, Wong T, Nouranifar R, Iyengar R, Landau EM (1995). Postsynaptic CAMP pathway gates early LTP in hippocampal CA1 region. Neuron.

[6] English JD, Sweatt JD (1997). A requirement for the mitogen-activated protein kinase cascade in hippocampal long term potentiation. J. Biol. Chem..

[7] Giese KP, Fedorov NB, Filipkowski RK, Silva AJ (1998). Autophosphorylation at Thr286 of the α calcium-calmodulin kinase II in LTP and learning. Science(80-.)..

[8] Colley PA, Sheu FS, Routtenberg A (1990). Inhibition of protein kinase C blocks two components of LTP persistence, leaving initial potentiation intact. J. Neurosci..

[9] Lopez-Molina L, Boddeke H, Muller D (1993). Blockade of long-term potentiation and of NMDA receptors by the protein kinase C antagonist calphostin C. Naunyn. Schmiedebergs. Arch. Pharmacol..

[10] Matthies H, Reymann KG (1993). Protein kinase a inhibitors prevent the maintenance of hippocampal long-term potentiation. NeuroReport.

[11] Frey U, Huang YY, Kandel ER (1993). Effects of cAMP simulate a late stage of LTP in hippocampal CA1 neurons. Science (80-.)..

[12] Huang YY, Kandel ER (1994). Recruitment of long-lasting and protein kinase A-dependent long-term potentiation in the CA1 region of hippocampus requires repeated tetanization. Learn. Mem..

[13] Otmakhov N, Griffith LC, Lisman JE (1997). Postsynaptic inhibitors of calcium/calmodulin-dependent protein kinase type II block induction but not maintenance of pairing-induced long-term potentiation. J. Neurosci..

[14] Chen HX, Otmakhov N, Strack S, Colbran RJ, Lisman JE (2001). Is persistent activity of calcium/calmodulin-dependent kinase required for the maintenance of LTP?. J. Neurophysiol..

[15] Serrano P, Yao Y, Sacktor TC (2005). Persistent phosphorylation by protein kinase Mζ maintains late-phase long-term potentiation. J. Neurosci..

[16] Serrano P (2008). PKMζ maintains spatial, instrumental, and classically conditioned long-term memories. PLoS Biol..

[17] Sacktor TC (2008). Chapter 2 PKMζ, LTP maintenance, and the dynamic molecular biology of memory storage. Prog. Brain Res..

[18] Hrabetova S, Sacktor TC (1996). Bidirectional regulation of protein kinase Mζ in the maintenance of long-term potentiation and long-term depression. J. Neurosci..

[19] Ling DSF (2002). Protein kinase Mζ is necessary and sufficient for LTP maintenance. Nat. Neurosci..

[20] Sajikumar S, Navakkode S, Sacktor TC, Frey JU (2005). Synaptic tagging and cross-tagging: The role of protein kinase Mζ in maintaining long-term potentiation but not long-term depression. J. Neurosci..

[21] Pastalkova E (2006). Storage of spatial information by the maintenance mechanism of LTP. Science (80-.)..

[22] Madroñal N, Gruart A, Sacktor TC, Delgado-García JM (2010). PKMζ inhibition reverses learning-induced increases in hippocampal synaptic strength and memory during trace eyeblink conditioning. PLoS ONE.

[23] Hardt O, Migues PV, Hastings M, Wong J, Nader K (2010). PKMζ maintains 1-day- and 6-day-old long-term object location but not object identity memory in dorsal hippocampus. Hippocampus.

[24] Shema R, Sacktor TC, Dudai Y (2007). Rapid erasure of long-term memory associations in the cortex by an inhibitor of PKMζ. Science (80-.)..

[25] Shema R, Hazvi S, Sacktor TC, Dudai Y (2009). Boundary conditions for the maintenance of memory by PKMζ in neocortex. Learn. Mem..

[26] Sacco T, Sacchetti B (2010). Role of secondary sensory cortices in emotional memory storage and retrieval in rats. Science (80-.)..

[27] von Kraus LM, Sacktor TC, Francis JT (2010). Erasing sensorimotor memories via PKMζ inhibition. PLoS ONE.

[28] Migues PV (2010). PKMζ maintains memories by regulating GluR2-dependent AMPA receptor trafficking. Nat. Neurosci..

[29] Volk LJ, Bachman JL, Johnson R, Yu Y, Huganir RL (2013). PKM-ζ is not required for hippocampal synaptic plasticity, learning and memory. Nature.

[30] Lee AM (2013). Prkcz null mice show normal learning and memory. Nature.

[31] Tsokas P (2016). Compensation for PKMζ in long-term potentiation and spatial long-term memory in mutant mice. Elife.

[32] Gámiz F, Gallo M (2011). Intra-amygdala ZIP injections impair the memory of learned active avoidance responses and attenuate conditioned taste-aversion acquisition in rats. Learn. Mem..

[33] Wang S, Sheng T, Ren S, Tian T, Lu W (2016). Distinct roles of PKCι/λ and PKMζ in the initiation and maintenance of hippocampal long-term potentiation and memory. Cell Rep..

[34] Frame S, Cohen P (2001). GSK3 takes centre stage more than 20 years after its discovery. Biochem. J..

[35] Grimes CA, Jope RS (2001). The multifaceted roles of glycogen synthase kinase 3β in cellular signaling. Prog. Neurobiol..

[36] Ali A, Hoeflich KP, Woodgett JR (2001). Glycogen synthase kinase-3: properties, functions, and regulation. Chem. Rev..

[37] Doble BW, Woodgett JR (2003). GSK-3: Tricks of the trade for a multi-tasking kinase. J. Cell Sci..

[38] Hooper C (2007). Glycogen synthase kinase-3 inhibition is integral to long-term potentiation. Eur. J. Neurosci..

[39] Ahmadian G (2004). Tyrosine phosphorylation of GluR2 is required for insulin-stimulated AMPA receptor endocytosis and LTD. EMBO J..

[40] Wei J, Liu W, Yan Z (2010). Regulation of AMPA receptor trafficking and function by glycogen synthase kinase 3. J. Biol. Chem..

[41] Giese KP (2009). GSK-3: A key player in neurodegeneration and memory. IUBMB Life.

[42] Frame S, Cohen P, Biondi RM (2001). A common phosphate binding site explains the unique substrate specificity of GSK3 and its inactivation by phosphorylation. Mol. Cell.

[43] Clem RL, Huganir RL (2010). Calcium-permeable AMPA receptor dynamics mediate fear memory erasure. Science.

[44] Díaz-Mataix L, Dȩbiec J, LeDoux JE, Doyère V (2011). Sensory-specific associations stored in the lateral amygdala allow for selective alteration of fear memories. J. Neurosci..

[45] Kim J (2007). Amygdala depotentiation and fear extinction. Proc. Natl. Acad. Sci. U. S. A..

[46] McKernan MG, Shinnick-Gallagher P (1997). Fear conditioning induces a lasting potentiation of synaptic currents in vitro. Nature.

[47] Rumpel S, LeDoux J, Zador A, Malinow R (2005). Postsynaptic receptor trafficking underlying a form of associative learning. Science (80-.)..

[48] Rogan MT, Staubli UV, LeDoux JE (1997). Fear conditioning induces associative long-term potentiation in the amygdala. Nature.

[49] Parsons RG, Davis M (2011). Temporary disruption of fear-potentiated startle following PKMζ. Nat. Neurosci..

[50] Nader K (2011). On the temporary nature of disruption of fear-potentiated startle following PKMζ inhibition in the amygdale. Front. Behav. Neurosci..

[51] Sacktor TC (2012). Memory maintenance by PKMζ—an evolutionary perspective. Mol. Brain.

[52] Parsons RG, Davis M (2011). Gone but not forgotten. Front. Behav. Neurosci..

[53] Chew B (2015). Lentiviral silencing of GSK-3β in adult dentate gyrus impairs contextual fear memory and synaptic plasticity. Front. Behav. Neurosci..

[54] Sadeh N, Verbitsky S, Dudai Y, Segal M (2015). Zeta inhibitory peptide, a candidate inhibitor of protein kinase Mζ, is excitotoxic to cultured hippocampal neurons. J. Neurosci..

[55] LeBlancq MJ, McKinney TL, Dickson CT (2016). ZIP It: neural silencing is an additional effect of the PKM-zeta inhibitor zeta-inhibitory peptide. J. Neurosci..

[56] Tovote P, Fadok JP, Lüthi A (2015). Neuronal circuits for fear and anxiety. Nat. Rev. Neurosci..

[57] Shabashov D, Shohami E, Yaka R (2012). Inactivation of PKMζ in the NAc shell abolished cocaine-conditioned reward. J. Mol. Neurosci..

[58] Wu-Zhang AX, Schramm CL, Nabavi S, Malinow R, Newton AC (2012). Cellular pharmacology of protein kinase Mζ (PKMζ) contrasts with its in vitro profile: Implications for PKMζ as a mediator of memory. J. Biol. Chem..

[59] Yao Y (2013). Matching biochemical and functional efficacies confirm ZIP as a potent competitive inhibitor of PKMζ in neurons. Neuropharmacology.

[60] Krotova K (2006). Peptides modified by myristoylation activate eNOS in endothelial cells through Akt phosphorylation. Br. J. Pharmacol..

[61] Lim S (2008). A myristoylated pseudosubstrate peptide of PKC-ζ induces degranulation in HMC-1 cells independently of PKC-ζ activity. Life Sci..

[62] Kawakami F, Yamaguchi A, Suzuki K, Yamamoto T, Ohtsuki K (2008). Biochemical characterization of phospholipids, sulfatide and heparin as potent stimulators for autophosphorylation of GSK-3β and the GSK-3β-mediated phosphorylation of myelin basic protein in vitro. J. Biochem..

[63] Peineau S (2007). LTP inhibits LTD in the hippocampus via regulation of GSK3β. Neuron.

[64] Zhu LQ (2007). Activation of glycogen synthase kinase-3 inhibits long-term potentiation with synapse-associated impairments. J. Neurosci..

[65] Kelly Á, Lynch MA (2000). Long-term potentiation in dentate gyrus of the rat is inhibited by the phosphoinositide 3-kinase inhibitor, wortmannin. Neuropharmacology.

[66] Sanna PP (2002). Phosphatidylinositol 3-kinase is required for the expression but not for the induction or the maintenance of long-term potentiation in the hippocampal CA1 region. J. Neurosci..

[67] Opazo P, Watabe AM, Grant SGN, O’Dell TJ (2003). Phosphatidylinositol 3-kinase regulates the induction of long-term potentiation through extracellular signal-related kinase-independent mechanisms. J. Neurosci..

[68] Hernández F, Borrell J, Guaza C, Avila J, Lucas JJ (2002). Spatial learning deficit in transgenic mice that conditionally over-express GSK-3β in the brain but do not form tau filaments. J. Neurochem..

[69] Liu SJ (2003). Overactivation of glycogen synthase kinase-3 by inhibition of phosphoinositol-3 kinase and protein kinase C leads to hyperphosphorylation of tau and impairment of spatial memory. J. Neurochem..

[70] Zhu Y (2005). Rap2-JNK removes synaptic AMPA receptors during depotentiation. Neuron.

[71] Leroy K (2002). The active form of glycogen synthase kinase-3β is associated with granulovacuolar degeneration in neurons in Alzheimers’s disease. Acta Neuropathol..

[72] Nagao M, Hayashi H (2009). Glycogen synthase kinase-3beta is associated with Parkinson’s disease. Neurosci. Lett..

[73] Karege F (2012). Protein levels of β-catenin and activation state of glycogen synthase kinase-3β in major depression. A study with postmortem prefrontal cortex. J. Affect. Disord..

[74] Polter A (2010). Deficiency in the inhibitory serine-phosphorylation of glycogen synthase kinase-3 increases sensitivity to mood disturbances. Neuropsychopharmacology.

[75] Emamian ES, Hall D, Birnbaum MJ, Karayiorgou M, Gogos JA (2004). Convergent evidence for impaired AKT1-GSK3β signaling in schizophrenia. Nat. Genet..

[76] Lee S, Kim J, Choi S (2018). Endogenous amyloid-β mediates memory forgetting in the normal brain. Biochem. Biophys. Res. Commun..

[77] Nelson CD, Kim MJ, Hsin H, Chen Y, Sheng M (2013). Phosphorylation of threonine-19 of PSD-95 by GSK-3β is required for PSD-95 mobilization and long-term depression. J. Neurosci..

[78] Llorens-Martín M (2013). GSK-3β overexpression causes reversible alterations on postsynaptic densities and dendritic morphology of hippocampal granule neurons in vivo. Mol. Psychiatry.

[79] Hong I (2013). AMPA receptor exchange underlies transient memory destabilization on retrieval. Proc. Natl. Acad. Sci. U. S. A..

[80] Rao-Ruiz P (2011). Retrieval-specific endocytosis of GluA2-AMPARs underlies adaptive reconsolidation of contextual fear. Nat. Neurosci..

[81] Migues PV (2016). Blocking synaptic removal of GluA2-containing AMPA receptors prevents the natural forgetting of long-term memories. J. Neurosci..

[82] Crispim Junior CF (2012). ETHOWATCHER: Validation of a tool for behavioral and video-tracking analysis in laboratory animals. Comput. Biol. Med..

[83] Choi S, Klingauf J, Tsien RW (2000). Postfusional regulation of cleft glutamate concentration during LTP at ‘silent synapses’. Nat. Neurosci..

[84] Hong I (2009). Extinction of cued fear memory involves a distinct form of depotentiation at cortical input synapses onto the lateral amygdala. Eur. J. Neurosci..

[85] Kim W, Won SY, Yoon BJ (2019). CRMP2 mediates GSK3β actions in the striatum on regulating neuronal structure and mania-like behavior. J. Affect. Disord..

[86] Kim J (2007). Blockade of amygdala metabotropic glutamate receptor subtype 1 impairs fear extinction. Biochem. Biophys. Res. Commun..

[87] Meijer L (2003). GSK-3-Selective inhibitors derived from Tyrian purple indirubins. Chem. Biol..

[88] Likhtik E, Popa D, Apergis-Schoute J, Fidacaro GA, Paré D (2008). Amygdala intercalated neurons are required for expression of fear extinction. Nature.

[89] An B (2017). Amount of fear extinction changes its underlying mechanisms. Elife.

[90] Lee S (2013). GluA1 phosphorylation at serine 831 in the lateral amygdala is required for fear renewal. Nat. Neurosci..

