# Supplementary material for: GSK-3β activation is required for ZIP-induced disruption of learned fear
Source: Sci Rep. 2020 Oct 26;10:18227. doi: 10.1038/s41598-020-75130-5 (PMC7588416; doi:10.1038/s41598-020-75130-5)
Supplement: Supplementary file 1 — Supplementary Information. [file 41598_2020_75130_MOESM1_ESM.docx]

**Supplementary Information**

**GSK-3β activation is required for ZIP-induced disruption of learned fear**

Sukwoon Song^1^, Jihye Kim^1^, Kyungjoon Park^3^, Junghwa Lee^1^, Sewon Park^1^, Sukwon Lee^2^, Jeongyeon Kim^2^, Ingie Hong^4^, Beomjong Song^5^, and Sukwoo Choi^1^

^1^ School of Biological Sciences, College of Natural Sciences, Seoul National University, Seoul, Korea (ROK)

^2^ Department of Neural Development and Disease, Korea Brain Research Institute, Daegu, Korea

^3^ Department of Psychiatry, McLean Hospital, Harvard Medical School, Belmont, MA 02478, USA

^4^ The Solomon H. Snyder Department of Neuroscience, Johns Hopkins University School of xMedicine, Baltimore, MD, USA

^5^ International Research Center for Neurointelligence (WPI-IRCN), The University of Tokyo Institutes for Advanced Study (UTIAS), The University of Tokyo, Tokyo, Japan

Correspondence: Ingie Hong (ingiehong@jhmi.edu), Beomjong Song (b_song@m.u-tokyo.ac.jp) or Sukwoo Choi (lead contact: sukwoo12@snu.ac.kr)

These authors contributed equally: Sukwoon Song, Jihye Kim, Kyungjoon Park, Junghwa Lee

**Supplementary Figure S1**

LTP stimuli

*

*

*

*****

**Supplementary Figure S2**

BIO-ZIP

BIO-Veh

BIO-ZIP

BIO-Veh

GSK-3β shRNA-Veh

GSK-3β shRNA-ZIP

Ctrl shRNA-ZIP

Ctrl shRNA-Veh

Veh-Veh

Veh-ZIP


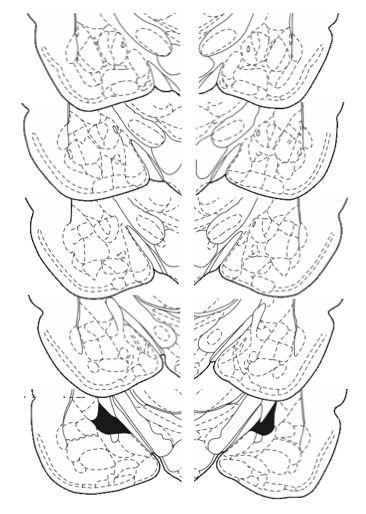


-2.30 mm

-2.56 mm

-2.80 mm

-3.30 mm

-3.60 mm


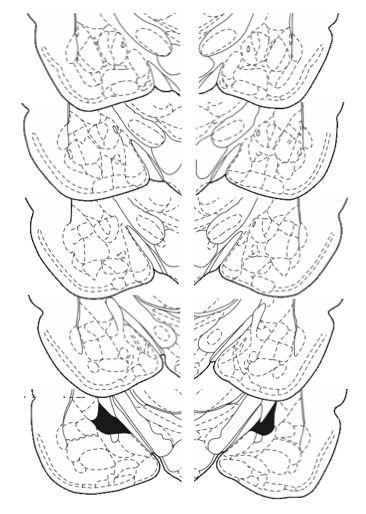


-2.30 mm

-2.56 mm

-2.80 mm

-3.30 mm

-3.60 mm


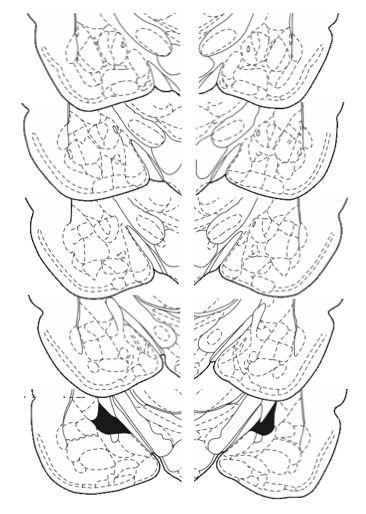


-2.30 mm

-2.56 mm

-2.80 mm

-3.30 mm

-3.60 mm


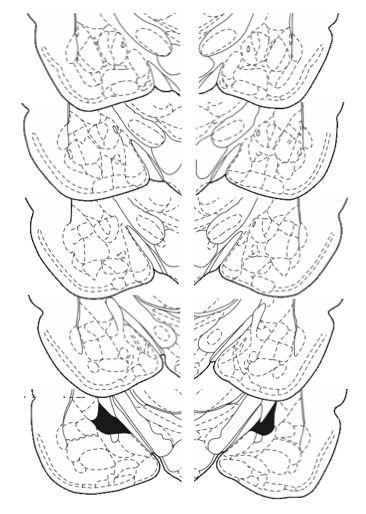


-2.30 mm

-2.56 mm

-2.80 mm

-3.30 mm

-3.60 mm


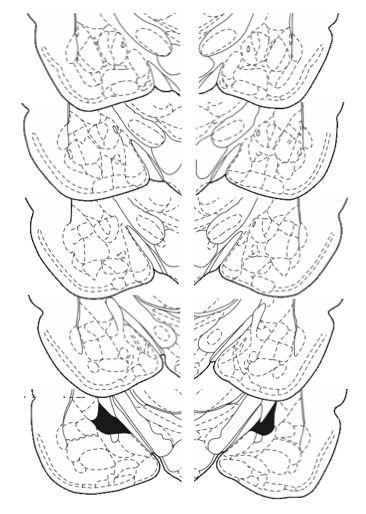


-2.30 mm

-2.56 mm

-2.80 mm

-3.30 mm

-3.60 mm


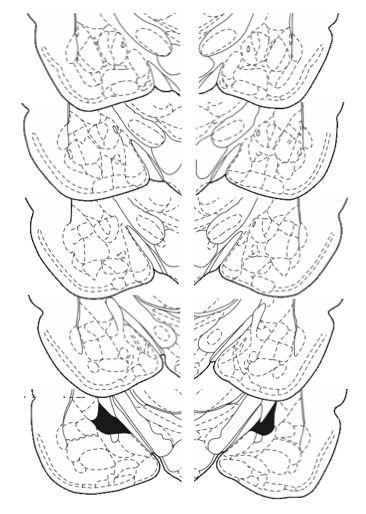


-2.30 mm

-2.56 mm

-2.80 mm

-3.30 mm

-3.60 mm

Veh-Veh

Veh-ZIP

**c**

**b**

**a**

**Supplementary Figure S3**

**a**

Total
fraction

Synaptosomal fraction

Synaptosomal fraction

**b**

Total
fraction

Standard

Standard

**
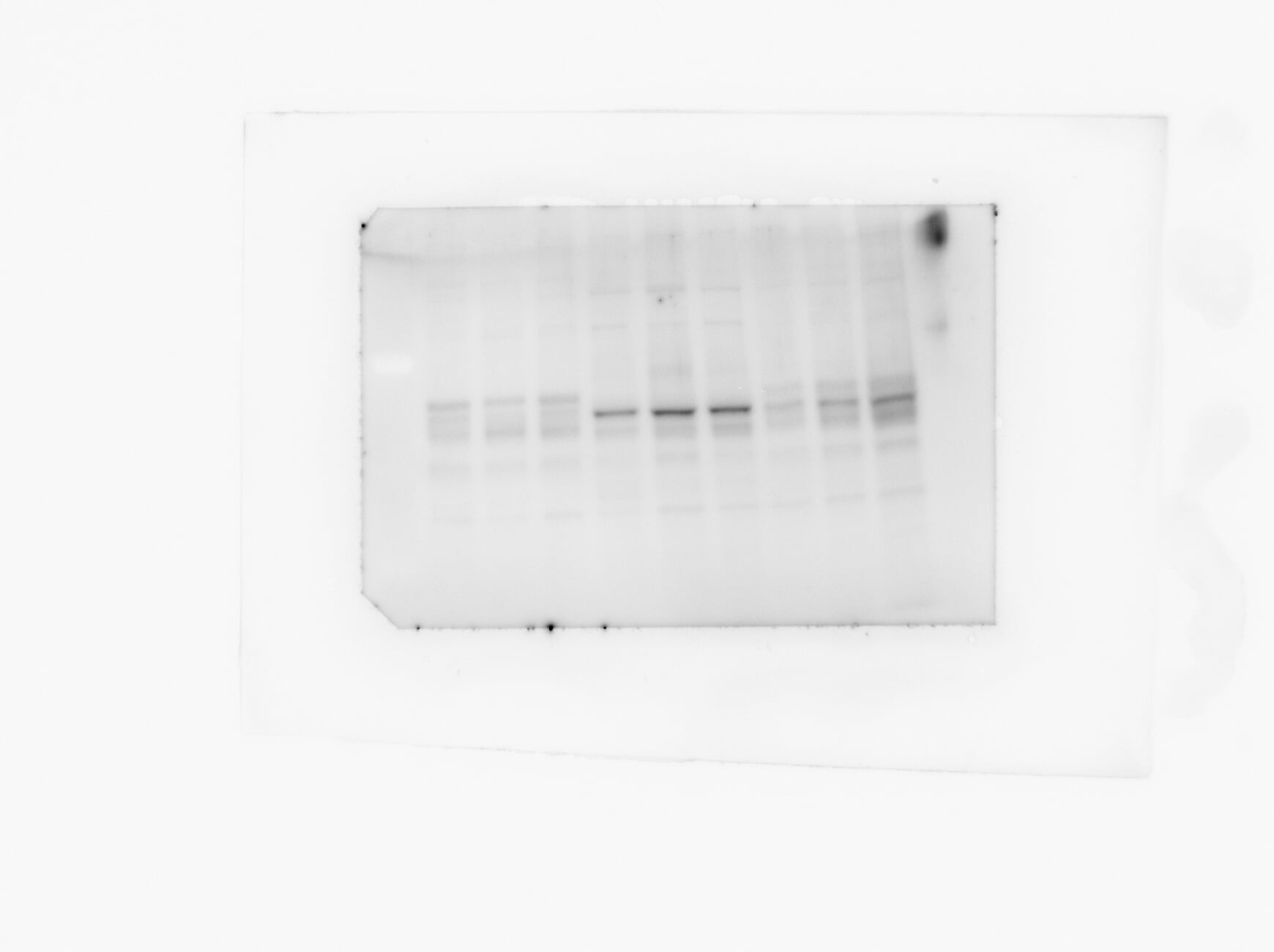

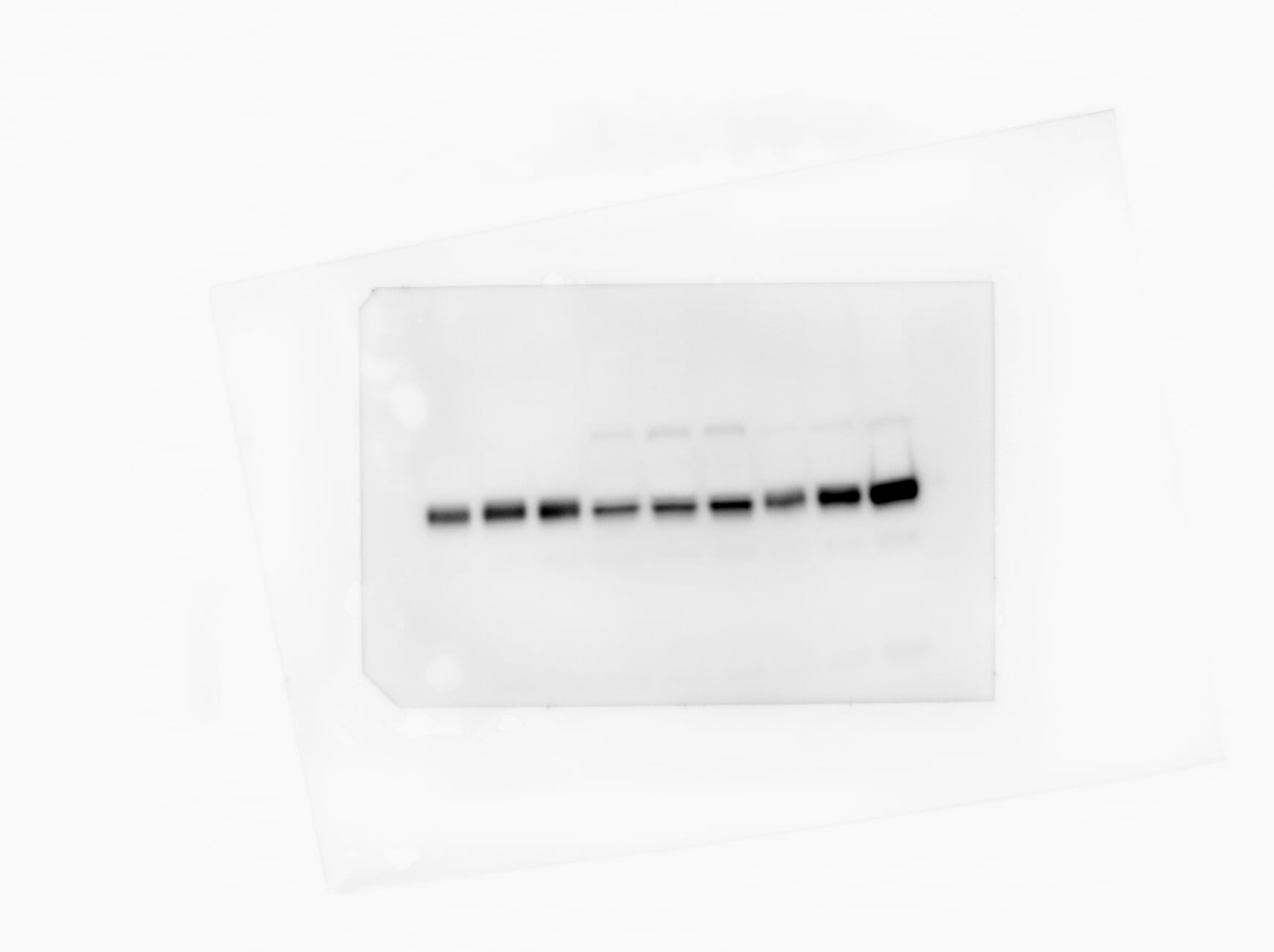
**

GSK-3α

GSK-3β

GSK-3β

Ctrl
shRNA

GSK-3β
shRNA


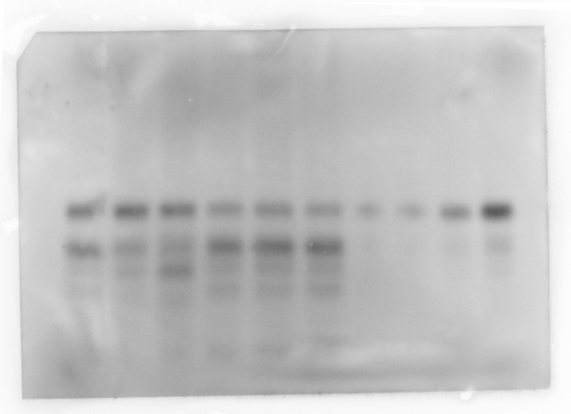


Standard


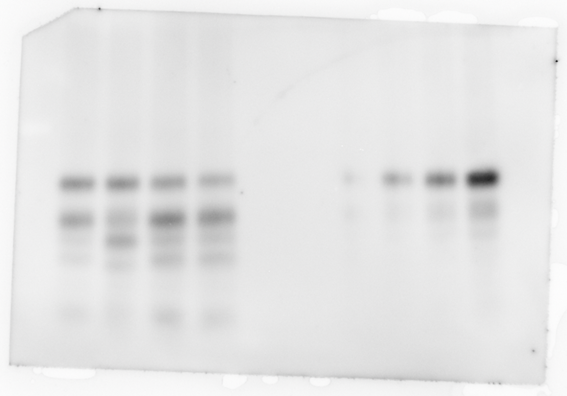


GSK-3β

Ctrl
shRNA

GSK-3β
shRNA

Standard

**c**

**d**


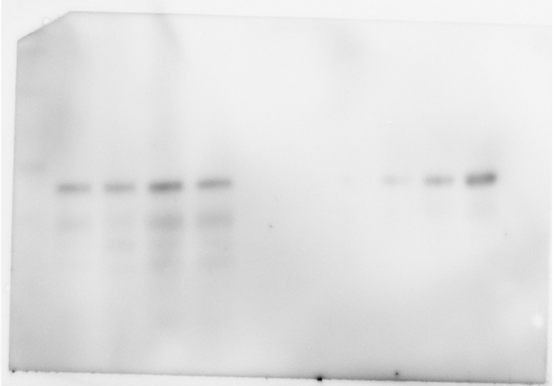


Actin

Ctrl
shRNA

GSK-3β
shRNA

Standard

**e**

**f**


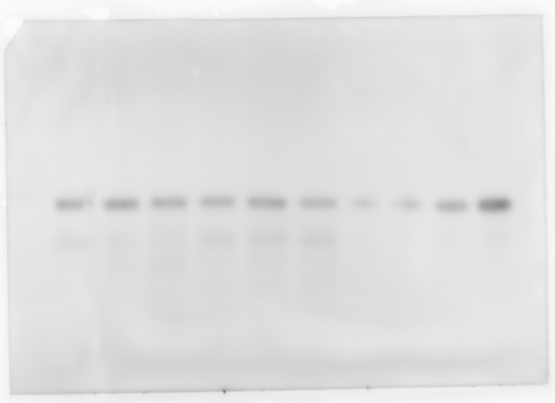


Actin

Ctrl
shRNA

GSK-3β
shRNA

Standard

**SUPPLEMENTARY INFORMATION**

**Figure S1. Blocking GSK-3β by BIO-acetoxime inhibited ZIP-induced depotentiation of late-phase LTP in slices prepared from naïve rats.**

Extracellular field potentials were measured in the LA while stimulating the thalamic input to the LA. Population spike (PS) amplitudes in response to the stimulation were plotted as a function of the recording time in two experimental groups (ZIP, n = 5; ZIP and BIO-acetoxime, n = 6). After recording 60 min of baseline, L-LTP was induced by six trains of high-frequency stimulation (HFS). ZIP (5 μM) or ZIP & BIO-acetoxime (0.3 μM) were applied 180 min after HFS. Traces from the last 30 min of the treatment were analyzed. Significant differences between ZIP and ZIP & BIO-acetoxime groups were observed (ZIP, 100.3 ± 8.550%; ZIP-BIO-acetoxime, 136.1 ± 11.24%, two-tailed t-test, p < 0.05, and the two groups passed the Kolmogorov-Smirnov normality test).

**Figure S2. Schematic representation of the injector cannula tips.** Histological plates illustrating the injection sites in the LA were adopted from the rat brain atlas (Paxinos, George, and Charles Watson. The rat brain in stereotaxic coordinates. Academic Press, 1997.). **(a)** Location of the injection cannula tips for the experiments shown in **Fig. 3b**. (**b)** Location of the injection cannula tips for the experiments shown in **Fig. 3c**. (**c)** Location of the injection cannula tips for the experiments shown in **Fig. 3f**

**Figure S3. (a, b)** Full-length blots from **Fig. 1d. (c-f)** Full-length blots from **Fig. 3e.** The red boxes indicate the regions which are displayed in the main figures.

**Supplementary methods**

**Field-Potential Recordings**

Extracellular field-potential recordings were performed using a parylene-insulated microelectrode (573210; A-M Systems, Sequim, WA, USA) in 400 mm-thick slices. Stimuli to thalamic pathways elicited simple negative field potentials that had a constant latency of ~4 ms and a duration of 5–15 ms. Baseline stimulation (0.017 Hz, 0.2 ms pulse duration) was delivered at an intensity (typically 10–25 mA) that evoked a response that was ≥50% of the maximum evoked response. A submersion-type recording chamber (≥0.5 mL in volume) was continuously superfused with aCSF (33.0–34.5°C) at a constant flow rate of 1–2 mL/min maintained by a peristaltic pump (Pharmacia). Extracellular field potentials were amplified and filtered (low-pass filter, 1 kHz; high-pass filter, 1 Hz; Dam80; World Precision Instruments), and then digitized at 1 kHz (ADC-42; Pico Technologies, Cambridgeshire, UK) or at 20 kHz (NAC 2.0 acquisition system, Theta Burst Corporation, Irvain, CA, USA). The digitized signals were stored and analyzed using the LTP program (www.LTP-program.com) or NAC Gather software. LTP was elicited by six trains of HFS (100 Hz, 1 s duration, 1 min interval) with the same intensity and pulse duration as the test stimuli. One or two slices were recorded per animal. To obtain stable, long-term recordings, we began recording at least 3.5 h after preparation of the 400 mm-thick slices. To improve the signal-to-noise ratio, data were averaged using a three-point running average in the time-lapse experiments.
